# Assessment of a Risk Index for Suicide Attempts Among US Army Soldiers With Suicide Ideation: Analysis of Data From the Army Study to Assess Risk and Resilience in Servicemembers (Army STARRS)

**DOI:** 10.1001/jamanetworkopen.2019.0766

**Published:** 2019-03-15

**Authors:** Kelly L. Zuromski, Samantha L. Bernecker, Peter M. Gutierrez, Thomas E. Joiner, Andrew J. King, Howard Liu, James A. Naifeh, Matthew K. Nock, Nancy A. Sampson, Alan M. Zaslavsky, Murray B. Stein, Robert J. Ursano, Ronald C. Kessler

**Affiliations:** 1 Department of Health Care Policy, Harvard Medical School, Boston, Massachusetts; 2 Department of Psychology, Harvard University, Cambridge, Massachusetts; 3 Department of Psychiatry, University of Colorado School of Medicine, Aurora; 4 Rocky Mountain Mental Illness Research, Education, and Clinical Center, Rocky Mountain Regional Veterans Affairs Medical Center, Aurora, Colorado; 5 Department of Psychology, Florida State University, Tallahassee; 6 Center for the Study of Traumatic Stress, Department of Psychiatry, Uniformed Services University School of Medicine, Bethesda, Maryland; 7 Department of Psychiatry, University of California, San Diego, La Jolla; 8 Department of Family Medicine and Public Health, University of California, San Diego, La Jolla

## Abstract

**Importance:**

The Department of Veterans Affairs recently began requiring annual suicide ideation (SI) screening of all patients and additional structured questions for patients reporting SI. Related changes are under consideration at the Department of Defense. These changes will presumably lead to higher SI detection, which will require hiring additional clinical staff and/or developing a clinical decision support system to focus in-depth suicide risk assessments on patients considered high risk.

**Objective:**

To carry out a proof-of-concept study for whether a brief structured question battery from a survey of US Army soldiers can help target in-depth suicide risk assessments by identifying soldiers with self-reported lifetime SI who are at highest risk of subsequent administratively recorded nonfatal suicide attempts (SAs).

**Design, Setting, and Participants:**

Cohort study with prospective observational design. Data were collected from May 2011 to February 2013. Participants were followed up through December 2014. Analyses were conducted from March to November 2018. A logistic regression model was used to assess risk for subsequent administratively recorded nonfatal SAs. A total of 3649 Regular Army soldiers in 3 Army Study to Assess Risk and Resilience in Servicemembers (Army STARRS) surveys who reported lifetime SI were followed up for 18 to 45 months from baseline to assess administratively reported nonfatal SAs.

**Main Outcomes and Measures:**

Outcome was administratively recorded nonfatal SAs between survey response and December 2014. Predictors were survey variables.

**Results:**

The 3649 survey respondents were 80.5% male and had a median (interquartile range) age of 29 (25-36) years (range, 18-55 years); 69.4% were white non-Hispanic, 14.6% were black, 9.0% were Hispanic, 7.0% were another racial/ethnic group. Sixty-five respondents had administratively recorded nonfatal SAs between survey response and December 2014. One additional respondent died by suicide without making a nonfatal SA but was excluded from analysis based on previous evidence that predictors are different for suicide death and nonfatal SAs. Significant risk factors were SI recency (odds ratio [OR], 7.2; 95% CI, 2.9-18.0) and persistence (OR, 2.6; 95% CI, 1.0-6.8), positive screens for mental disorders (OR, 26.2; 95% CI, 6.1-112.0), and Army career characteristics (OR for junior enlisted rank, 30.0; 95% CI, 3.3-272.5 and OR for senior enlisted rank, 6.7; 95% CI, 0.8-54.9). Cross-validated area under the curve was 0.78. The 10% of respondents with highest estimated risk accounted for 39.2% of subsequent SAs.

**Conclusions and Relevance:**

Results suggest the feasibility of developing a clinically useful risk index for SA among soldiers with SI using a small number of self-report questions. If implemented, a continuous quality improvement approach should be taken to refine the structured question series.

## Key Points

**Question:**

Is a short self-report battery associated with improved assessment of risk for suicide attempt among soldiers with suicide ideation?

**Findings:**

This cohort study of 3649 soldiers participating in the Army Study to Assess Risk and Resilience in Servicemembers survey found that a cross-validated model including self-reported history and severity of suicidal thoughts and behaviors, positive screens for mental disorders, and Army career characteristics was associated with administratively reported suicide attempts 18 to 45 months following baseline among respondents with lifetime suicide ideation at baseline. The 10% of those with suicide ideation who had the highest estimated risk accounted for 39.2% of subsequent suicide attempts.

**Meaning:**

It may be feasible to develop a clinical risk index for suicide attempt given suicide ideation from a small number of self-report questions.

## Teaser

This cohort study investigates whether a brief self-report battery from a survey of US Army soldiers is associated with identification of those with suicide ideation who are at the highest risk of suicide attempt.

## Introduction

The Department of Veterans Affairs/Department of Defense (VA/DoD) Clinical Practice Guidelines for the Assessment and Management of Patients at Risk of Suicide are currently being revised for release in 2019. In addition, the VA recently began implementing a new Suicide Risk Identification Strategy that includes an annual 3-stage suicide risk screening process for all VA health care patients.^1^ The DoD is considering related changes. This could lead to a substantial increase in the number of patients who are required to receive an in-depth suicide risk assessment. Such an assessment can be resource intensive, taking up to 2 hours per patient to complete.^2^

Two options exist to address possible increased demand for in-depth suicide risk assessments: hire additional clinical staff and/or develop a clinical decision support system to improve targeting of these assessments. With regard to the second option, although risk of suicidal behaviors is significantly elevated among people with suicide ideation (SI), suicidal behaviors are rare even in this high-risk segment of the population.^3,4^ Structured risk factor studies for suicidal behaviors among patients with SI typically conclude that prediction accuracy, while significantly greater than chance, is insufficient to guide clinical decision making because of the low proportion of high-risk patients who experience this rare outcome.^5^ However, it might be feasible to use a multistep decision-making process in which a brief initial battery of self-report questions administered to all patients reporting SI is used to develop a risk assessment model that isolates only a relatively small proportion of patients who have elevated risk of subsequent suicidal behaviors, allowing information about future risk to be used to help determine which patients would benefit most from in-depth suicide risk assessments added to usual care.^6^

Consistent with this possibility, a recent Army Study to Assess Risk and Resilience in Servicemembers (Army STARRS) study^7^ of self-reported suicide attempts (SAs) among soldiers with SI in a STARRS survey found that the 10% of soldiers with highest SA risk based on a composite risk index with a relatively small number of self-report survey risk factors accounted for more than 80% of all SAs among respondents with SI. However, the validity of this index is uncertain because it was based on retrospective reports in a cross-sectional survey, a common limitation in SA risk factor studies.^8^

The objective of the current report is to address this limitation by carrying out a prospective proof-of-concept study where information from a baseline survey of Army STARRS respondents is used to assess subsequent risk of administratively reported SAs. Although the survey was not designed with this purpose in mind, results could be valuable in providing a lower-bound estimate of the extent to which a brief structured question battery might be used to target in-depth suicide risk assessments by identifying patients with SI who are at high risk of subsequent SAs. The analysis was carried out in the same sample as the one used to create the retrospective risk index mentioned above, but with a focus on prospective administratively recorded SAs rather than retrospectively self-reported SAs.

## Methods

### Sample

The study followed the Strengthening the Reporting of Observational Studies in Epidemiology (STROBE) reporting guideline.^9^ Analysis was carried out in the Army STARRS Consolidated All Army Survey (AAS), an epidemiological study that combined data across 3 surveys:

1. The core AAS, a survey administered from 2011 to 2012 in a probability sample of 17 462 active Regular Army, National Guard, and Army Reserve units worldwide, excluding soldiers deployed or in basic training;
2. A 2012 to 2013 AAS expansion that surveyed 3987 deployed soldiers stationed in Afghanistan while in Kuwait awaiting transit to or from middeployment leave; and
3. The baseline STARRS Pre-Post Deployment Survey, which surveyed 8558 soldiers in 3 Brigade Combat Teams shortly before deploying to Afghanistan in 2012.

Recruitment, informed consent, and data collection procedures, described in more detail elsewhere,^10^ were approved by the Human Subjects Committees of the Uniformed Services University of the Health Sciences (Bethesda, Maryland) for the Henry M. Jackson Foundation (the primary grantee), the Institute for Social Research at the University of Michigan (Ann Arbor, the organization collecting the data), Harvard Medical School (Boston, Massachusetts), and University of California, San Diego (La Jolla). All study participants gave written informed consent. Analyses for the current article were conducted from March through November 2018.

Information on handling bias, including sample weighting, response rate, and sample exclusions, is reported elsewhere.^11^ We focus on the 27 501 Regular Army respondents with known survey dates. We excluded survey respondents who were in the activated Reserve Command because the administrative records needed to learn of SAs were missing for these individuals once they deactivated.

### Outcome

Prospective information about SA through December 2014 (18-45 months after survey participation) was assessed in Army/DoD administrative systems that together contain comprehensive health care encounter information. One of these, the DoD Suicide Event Report, is a standardized reporting system that defines SAs rigorously. However, this system underreports because it relies on active surveillance by local mental health professionals that sometimes does not occur, leading us to also include SAs defined by *International Classification of Diseases, Ninth Revision, Clinical Modification* codes E950 to E958.^12^ Combining the 2 definitions is justified by analyses reported elsewhere showing that they have very similar correlates.^12^ Soldiers who died by suicide but without a prior nonfatal SA were excluded from analysis based on evidence in previous research that the risk factors for suicide death are different from the risk factors for nonfatal SA.^8^

### Risk Factors

We focused on AAS respondents who reported either active (Did you ever in your life have thoughts of killing yourself?) or passive (Did you ever wish you were dead or would go to sleep and never wake up?) lifetime SI in the Columbia Suicidal Severity Rating Scale.^13^ Three broad classes of risk factors were considered based on results of our previous retrospective study.^7^

#### History and Severity of Self-Injurious Thoughts and Behaviors

Respondents reporting lifetime SI were asked about age of onset, years since onset, past 30-day SI, persistence during the week when SI was worst, and controllability during that week (How easy was it for you to control these thoughts or push them out of your mind when you wanted to?). Respondents were also asked about lifetime suicide plans and attempts, history of nonsuicidal self-injury, and history of “tempting fate” by engaging in dangerous behaviors that might have led to death.

#### Mental Disorders

The survey screened for 8 *Diagnostic and Statistical Manual of Mental Disorders, Fourth Edition,* mental disorders: lifetime major depressive episode, generalized anxiety disorder, posttraumatic stress disorder, bipolar disorder, panic disorder, intermittent explosive disorder, substance use disorders, and 6-month attention-deficit/hyperactivity disorder. The Composite International Diagnostic Interview^14^ screening scales^15,16^ were used to assess bipolar disorder, panic disorder, intermittent explosive disorder, and attention-deficit/hyperactivity disorder. A modified self-report Family History Screen^17^ was used to screen for the other disorders. Diagnoses based on the Composite International Diagnostic Interview and Family History Screen have demonstrated good concordance with diagnoses based on blinded clinical interviews.^15,16,17^

#### Sociodemographic and Army Career Characteristics

To consider demographic differences associated with SA, we also considered several sociodemographic variables (age, sex, race/ethnicity [categories defined by Army STARRS team and self-identified by participants], and marital status) and Army career characteristics (years in service, rank [junior enlisted E1-E4, senior enlisted E5-E9, officers including Warrant officers], deployment history, and military occupational specialty [using broad categories of combat arms, combat support, and combat service support]). More details on military occupational specialty are reported elsewhere.^7^

### Statistical Analysis

Prior to conducting primary analyses, 20 multiple imputation (MI) person-month samples were constructed, each containing all 810 181 person-months across all respondents between the month of survey and either the month of first subsequent administratively recorded SA, month of separation from service, month of death, or December 2014, whichever came first. Cross-tabulations were used in this MI data set to examine associations of lifetime SI as of the time of survey with subsequent SA.

To build a model assessing risk of SA given lifetime SI, we used discrete-time person-month survival analysis with a logistic link function^18^ in the subsample of the 20 MI samples made up of respondents who reported lifetime SI in the survey (102 231 person-months). All survival analyses were conducted in SAS statistical software (SAS Institute Inc). Case-control subsampling was used for computational efficiency including all person-months with an SA and a probability sample of 20 times as many control person-months weighted by the inverse of their probability of selection. We considered all of the categories of risk factor variables listed above (ie, history and severity of self-injurious thoughts and behaviors, mental disorders, and sociodemographic and Army career characteristics) in analyses.

Univariate risk factors that were significantly associated with SA in this MI data set were combined to generate multivariate models within each of these 3 broad risk factor categories (eTables 1-4 in the Supplement). These models were then trimmed of nonsignificant risk factors, and remaining within-category risk factors were included in a final combined model. Logits ± 2 times MI standard errors taking the weighting and clustering of the samples into consideration using the Taylor series method^19^ were exponentiated to produce odds ratios (ORs) and 95% confidence intervals. We adjusted for overfitting in estimating out-of-sample performance by using 10-fold cross-validation (10F-CV)^20^ in each of the 20 MI data sets to generate a pooled receiver operating characteristic (ROC) curve.^21^ Area under the ROC curve (AUC) was calculated to evaluate model fit.

Given that some theories of risk factors for suicide hypothesize the existence of interactions among some of the risk factors we considered,^22^ we also used the Super Learner ensemble machine learning algorithm^23^ in R statistical software (R Project for Statistical Computing) to investigate whether stable interactions existed among risk factors included in our SA risk index. Super Learner creates optimal weights to combine associations based on a library of different machine learning classifiers through model averaging. Thirty-two classifers were used in our library (eTable 5 in the Supplement). Twenty replicates of 5F-CV were used to generate an ROC curve based on the Super Learner ensemble. We used 5F-CV rather than the 10F-CV used in the logistic model because Super Learner already uses internal 10F-CV to estimate models and develop the weights combining results across classifiers. Risk strata based on inspection of the cross-validated ROC curves were created^24^ for purposes of calculating positive predictive value (expressed as the number of observed SAs per 100 000 person-years) and sensitivity (proportion of observed SAs among people in each risk stratum).

For all statistical tests conducted, we used 2-sided hypothesis tests and an a priori .05 level of significance. Missing data, which were very uncommon, were recoded to medians for all variables other than several aspects of self-injurious behaviors (missing in the range of 4%-12% across predictors). The latter were imputed using the method of MI^25^ with 20 MI replicates generated using SAS proc MI.^26^

## Results

The Army STARRS Consolidated AAS sample consisted of 27 501 Regular Army soldiers (86.6% male; median [interquartile range] age as of December 2014, 27 [22-34] years [range, 18-68 years]; 64.1% white non-Hispanic, 17.3% black, 11.5% Hispanic, 7.1% other; 34.4% currently married, 59.1% never married, 6.5% previously married). Most of our analyses focused on the subset of Consolidated AAS respondents who endorsed lifetime SI (14.3% [3649 respondents]; 80.5% male; median [interquartile range] age as of December 2014, 29 [25-36] years [range, 18-55 years]; 69.4% white non-Hispanic, 14.6% black, 9.0% Hispanic, 7.0% other; 65.7% currently married, 25.1% never married, 9.2% previously married).

### Distribution of Prospective SAs by Baseline SI History

From the full AAS sample, a weighted 14.3% of survey respondents reported lifetime SI, 7.5% without a lifetime plan or SA, 3.8% with a lifetime plan but no SA, 0.9% with a history of unplanned SA (ie, those who endorsed a prior SA but denied prior suicide plans), and 2.1% with a history of planned SA (ie, those who endorsed both prior SA and prior suicide plans) (Table 1). Subsequent administratively recorded SAs occurred from 18 to 45 months after baseline among a weighted 0.7% of AAS respondents (220 participants). This baseline suicidality gradient was significantly associated with subsequent SAs (χ^2^_4_ = 9.4; *P* < .001). Prospective SA risk was lowest among soldiers with no lifetime SI in the AAS (236.4/100 000 person-years), nonsignificantly higher among soldiers with lifetime SI but no prior plan or SA (360.0/100 000 person-years; OR, 1.4; 95% CI, 0.7-3.1), and significantly higher among soldiers with a lifetime plan but no prior SA (529.2/100 000 person-years; OR, 3.1; 95% CI, 1.2-7.8), a prior unplanned SA (616.8/100 000 person-years; OR, 4.2; 95% CI, 1.1-15.4), and a prior planned SA (1202.4/100 000 person-years; OR, 8.8; 95% CI, 4.2-18.7).

**Table 1.** Distribution and Associations of Survey Self-Reported Lifetime Suicidality With Subsequent SAs in 27 501 Participants^a^

Survey-Reported Lifetime Suicidality	Distribution of Survey-Reported Lifetime Suicidality (n = 27 501)		Prospective SAs			
			Distribution of Prospective SAs (n = 220)		SAs/100 000 Person-Years^c^	OR (95% CI)
	% (SE)	No.^b^	% (SE)	No.^b^		
No lifetime suicidality	85.7 (0.3)	23 852	73.9 (4.2)	155	236.4 (25.2)	1 [Reference]
Any lifetime suicidality	14.3 (0.3)	3649	26.1 (4.2)	65	536.4 (98.4)	3.0 (1.8-5.0)^d^
Ideation only	7.5 (0.3)	2118	9.6 (3.0)	29	360.0 (111.6)	1.4 (0.7-3.1)
Ideation with a plan but no attempt	3.8 (0.2)	787	6.7 (2.4)	11	529.2 (204.0)	3.1 (1.2-7.8)^d^
Ideation with an unplanned attempt^e^	0.9 (0.1)	219	1.6 (0.8)	6	616.8 (342.0)	4.2 (1.1-15.4)^d^
Ideation with a planned attempt^f^	2.1 (0.2)	525	8.3 (2.3)	19	1202.4 (369.6)	8.8 (4.2-18.7)^d^
*F*_4_^g^						9.4^d^

Abbreviations: OR, odds ratio; SA, suicide attempt.

^a^ Results for all Regular Army soldiers in the Army Study to Assess Risk and Resilience in Servicemembers Consolidated All Army Survey Results reflect weighted and multiply imputed data.

^b^ Percentage is based on weighted data but number is unweighted.

^c^ Respondents with SAs were censored at the month of their first SA.

^d^ Significant at the .05 level, 2-sided multiply imputed adjusted test.

^e^ Respondents reported a lifetime SA in the survey but denied ever having a suicide plan.

^f^ Respondents reported a lifetime SA in the survey with a suicide plan.

^g^ *F* test to evaluate the joint significance of the association between survey lifetime suicidality and subsequent administratively recorded nonfatal SAs.

### Risk Factors Associated With Administratively Recorded SAs Among Soldiers With Prior Lifetime SI

Further analysis focused on the 14.3% of Regular Army AAS respondents with lifetime SI (3649 participants), who accounted for 26.1% of subsequent administratively recorded definite or probable SAs (65 respondents made 77 SAs consisting of 22 poisonings, 16 alcohol or drug related, 12 blunt or sharp objects, 8 firearm related, 12 other methods, and 7 missing method). Four other aspects of baseline suicidality (eTable 1 and eTable 2 in the Supplement) were significantly associated with SA in univariate survival models: SI recency in the 30 days before survey, persistence of worst-week SI, lifetime plan, and 2 or more prior lifetime SAs. Other risk factors significant at the univariate level were positive screens for 2 lifetime mental disorders (posttraumatic stress disorder and attention-deficit/hyperactivity disorder), counts for number of lifetime internalizing, externalizing, and total disorder screens (eTable 3 in the Supplement), and 3 highly intercorrelated measures of sociodemographic and career characteristics (young age, short time in service, and low enlisted rank) (eTable 4 in the Supplement). Four of these 8 significant risk factors remained significant in a final multivariate logistic model: 30-day SI (OR, 7.2; 95% CI, 2.9-18.0), worst-week SI persistence (OR, 2.6; 95% CI, 1.0-6.8), positive screens for 2 or more lifetime mental disorders (OR, 26.2; 95% CI, 6.1-112.0), and enlisted rank (OR for junior enlisted, 30.0; 95% CI, 3.3-272.5 and OR for senior enlisted, 6.7; 95% CI, 0.8-54.9) (Table 2). The cross-validated AUC of the final logistic model assessing subsequent risk of SA was 0.78. The cross-validated AUC of the Super Learner ensemble model using the same risk factors was 0.73, indicating that allowing for interactions did not improve assessment of SA risk compared with the main effects model.

**Table 2.** Associations of Survey Variables With Subsequent Suicide Attempts Given Survey-Reported Lifetime Suicide Ideation in 3649 Participants^a^

Variable	Distribution of Survey Variables^b^		Best-Fitting Multivariate Logistic Model, OR (95% CI)^c^
	% (SE)	No.	
History of suicidal thoughts and behaviors			
Ideation onset at age 15-17 y vs ≤14 y	20.2 (1.0)	721	1.8 (0.6-5.5)
Ideation onset at age ≥18 y vs ≤14 y	46.2 (1.3)	1632	2.4 (0.6-10.2)
*F*_2_^d^			0.9
*F*_1_^e^			0.2
Time since onset of ideation, mean (SD), y^f^	7.6 (3.0)	NA	1.1 (0.9-1.3)
Active ideation vs passive	79.0 (1.1)	2927	1.1 (0.2-4.8)
Ideation within past 30 d	10.5 (0.8)	426	7.2 (2.9-18.0)^g^
Mental disorders			
Count ≥2 vs 0 or 1	78.6 (1.4)	2675	26.2 (6.1-112.0)^g^
Worst-week ideation persistence			
7 d and/or ≥9 h/d vs neither	29.0 (1.0)	1054	2.6 (1.0-6.8)^g^
Sociodemographic and Army career variables			
Rank: junior vs officer	45.7 (1.5)	1894	30.0 (3.3-272.5)^g^
Rank: senior vs officer	38.1 (1.6)	1335	6.7 (0.8-54.9)
*F*_2_^d^			7.0^g^
*F*_1_^e^			9.2^g^

Abbreviation: OR, odds ratio.

^a^ Results for Regular Army soldiers in the Army Study to Assess Risk and Resilience in Servicemembers Consolidated All Army Survey who self-reported lifetime suicide ideation. Results reflect weighted and multiply imputed data.

^b^ Percentage is based on weighted data but number is unweighted.

^c^ The best-fitting multivariate model dropped nonsignificant survey variables from prior models for each category of such variables (see eTables 1-4 in the Supplement) other than controls for ideation age at onset, years since ideation age at onset, and active (vs passive) ideation.

^d^ *F* test to evaluate the joint significance of categorical variable levels.

^e^ *F* test to evaluate whether the ORs for this categorical variable are significantly different from each other.

^f^ Time since onset of suicide ideation ranged from 1 to 11 years, with values top coded at 11 (ie, if a participant reported ideation onset 15 years ago, they were set to 11).

^g^ Significant at the .05 level, 2-sided multiply imputed adjusted test.

### Model Performance

Risk strata were collapsed based on inspection of cross-validated ROC curves (Figure). The proportion of observed SAs among the 5% and 10% of AAS respondents in the highest-risk strata (sensitivity) was much higher than the 5% to 10% expected by chance (33.5%-39.2%) (Table 3). Suicide attempts occurred at a rate of 3962.4/100 000 person-years in the highest-risk ventile (positive predictive value). The 30% of soldiers with lowest risk accounted for 7.3% of SAs.

**Figure.**
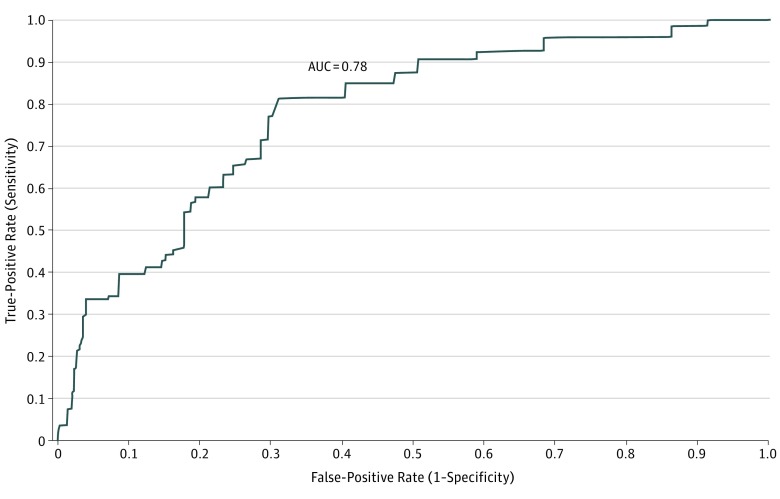
Receiver Operating Characteristic Curve of 10-Fold Cross-Validated Logistic Model Predicting Subsequent Suicide Attempts Given Survey-Reported Lifetime Suicide Ideation in 3649 Participants The receiver operating characteristic curve estimates out-of-sample performance of the final logistic regression model (based on 20 replicates of 10-fold cross-validation) with subsequent administratively recorded suicide attempts as the outcome among Regular Army soldiers who reported lifetime suicide ideation in the Army Study to Assess Risk and Resilience in Servicemembers Consolidated All Army Survey. AUC indicates area under the curve.

**Table 3.** Associations of 10-Fold Cross-Validated Risk Strata With Subsequent Suicide Attempts Given Survey-Reported Lifetime Suicidality in 3649 Participants^a^

Percentile	Sensitivity, % (SE)^b^	PPV, No. (SE)^c^	Alternative Contrasts Among Risk Strata, OR (95% CI)	
			5 Strata	3 Strata
Top 10%	39.2 (8.2)	2244.4 (618.8)		9.4 (4.5-19.4)^d^
Top 5%	33.5 (7.8)	3.692.4 (1071.3)	32.9 (10.4-103.8)^d^	
6%-10%	5.7 (4.4)	633.6 (514.6)	5.11 (0.7-37.9)	
11%-30%	28.7 (8.9)	824.5 (306.1)	6.8 (1.8-26.6)^d^	3.4 (1.3-8.8)^d^
Bottom 70%	32.1 (8.0)	239.7 (67.3)		1 [Reference]
31%-70%	24.8 (7.6)	338.7 (107.7)	2.8 (0.8-10.3)	
71%-100%	7.3 (4.1)	120.3 (70.1)	1.0 [Reference]	
*F*_4/2_^e^			16.0^d^	18.0^d^

Abbreviations: OR, odds ratio; PPV, positive predictive value.

^a^ Results for Regular Army soldiers in the Army Study to Assess Risk and Resilience in Servicemembers Consolidated All Army Survey who self-reported lifetime suicide ideation. Estimates are pooled across 20 multiply imputed replicate data sets, each using 10-fold cross-validation.

^b^ Sensitivity is the proportion of all suicide attempts occurring among respondents in the risk stratum represented in the row.

^c^ Positive predictive value is the number of suicide attempts occurring per 100 000 person-years among respondents in the risk stratum represented in the row.

^d^ Significant at the .05 level, 2-sided multiply imputed adjusted test.

^e^ *F* test to evaluate the joint significance of categorical variable levels for alternative contrasts. The 4 *df F* test evaluates the significance of the 5-strata classification; the 2 *df F* test evaluates the significance of the 3-strata classification.

## Discussion

Consistent with previous research,^3,4,27^ we found that although SI is significantly associated with SA, only a minority (26.1%) of subsequent SAs occurred among the 14.3% of soldiers who reported a lifetime history of SI. Nonetheless, a risk classification system for soldiers with lifetime SI could be useful given their elevated SA risk as long as a parallel system is developed for soldiers who deny SI.^28^

Our cross-validated logistic regression model isolated 5% to 10% of respondents who accounted for substantially higher proportions of subsequent administratively recorded SAs given SI than expected by chance (33.5%-39.2%). The risk factors in this model were broadly consistent with those found in previous studies.^7,29,30,31^ In contrast to other recent Army STARRS work,^28^ we found no advantage to using a machine learning model that allowed for interactions over a simple logistic regression model, although meaningful interactions might have existed if our risk factor set was more comprehensive. That possibility needs to be considered if this line of investigation is pursued in the future.

A risk classification scheme with the strength of the one developed here would presumably not be of value in guiding decisions about care in the absence of a precision treatment model that found the classification scheme useful in predicting heterogeneity of treatment response because few patients screened as high risk will, in fact, make an SA, and most of those who made an SA were not classified as high risk.^5^ However, such a scheme, if improved (discussed below), could have considerable value in providing decision support for decisions regarding allocation of clinical resources. In addition, if the value of a true-positive relative to the cost of a false-positive can be specified precisely (including the cost of administering an in-depth suicide risk assessment to a patient who would not otherwise make an SA relative to the investment of that clinical effort in providing more intensive treatment for patients at high SA risk), an optimal decision threshold could be specified for such a scheme rather than using the arbitrary 5%, 10%, 30%, and 70% thresholds we used here.^32^ The development of this type of risk classification scheme aligns with decision tools used in other areas of medicine to weigh the relative costs and benefits of alternative clinical decisions (eg, Gage et al^33^).

It is noteworthy that the out-of-sample prediction accuracy of a model like the one developed here could be considerably higher than our cross-validated AUC because some known risk factors for SA were not included in our survey, such as frequency of 30-day SI, 30-day suicide plans, and intent to act on such plans.^4,34,35^ Nor did we include variables from electronic medical records, which have been shown to be associated with suicidal behaviors over and above self-report data.^36^ Furthermore, prediction accuracy would almost certainly have increased if we had focused on a shorter and more clinically useful time horizon than the 18 to 45 months we used here. We were forced to use this long time horizon because of the small sample size. Based on these considerations, it is reasonable to assume that prediction accuracy could be improved, perhaps substantially so, if a revised version of our data collection, assessment, and prediction scheme was implemented in a continuous quality improvement framework whereby exploratory data capture methods and questions were revised across successive revisions.^37^

### Limitations

Our results should be interpreted in the context of several limitations in addition to those involving question selection and time horizon. First, a population sample rather than a clinical sample was studied. Second, respondents were given assurance that their survey responses would be confidential, a guarantee not made by the military medical system. Third, we focused only on SAs recorded in administrative records. Methodological research suggests that the vast majority of such cases are, in fact, true SAs but that medical records miss other true SAs that are incorrectly coded as accidents in addition to those that never come to medical attention.^38,39,40^ These other SAs should be included if this line of investigation is pursued in the future.

## Conclusions

The high cross-validated concentration of risk of our best model (ie, 39.2% of all administratively recorded SAs occurring among the 10% of soldiers at highest predicted risk) suggests that a useful risk index could be developed that assigns a predicted SA risk score to all patients who report prior SI. In making successive refinements to create an optimal index, it would be useful to consider other self-report measures, emerging biomarkers, other novel measures (eg, natural language analysis of electronic clinical notes^41^ or social media posts^42^), and shorter time horizons for SA risk.

## Notes

Supplement. **eTable 1.** Self-Reported Risk Factors for Subsequent Administratively Recorded Suicide Attempts Involving History of Self-Injurious Thoughts and Behaviors Among Regular Army Soldiers Who Reported Lifetime Suicide Ideation in the STARRS Consolidated All Army Survey (n = 3,649) **eTable 2.** Self-Reported Risk Factors of Subsequent Administratively Recorded Suicide Attempts Involving Severity of Self-Injurious Thoughts and Behaviors Among Regular Army Soldiers Who Reported Lifetime Suicide Ideation in the STARRS Consolidated All Army Survey (n = 3,649) **eTable 3.** Self-Reported Risk Factors of Subsequent Administratively Recorded Suicide Attempts Involving History of Mental Disorders Among Regular Army Soldiers Who Reported Lifetime Suicide Ideation in the STARRS Consolidated All Army Survey (n = 3,649) **eTable 4.** Self-Reported Risk Factors of Subsequent Administratively Recorded Suicide Attempts Involving Socio-Demographics and Army Career Characteristics Among Regular Army Soldiers Who Reported Lifetime Suicide Ideation in the STARRS Consolidated All Army Survey (n = 3,649) **eTable 5.** Hyperparameter Settings for Super Learner Ensemble
