# Supplementary material for: Assessment of a Risk Index for Suicide Attempts Among US Army Soldiers With Suicide Ideation: Analysis of Data From the Army Study to Assess Risk and Resilience in Servicemembers (Army STARRS)
Source: JAMA Netw Open. 2019 Mar 15;2(3):e190766. doi: 10.1001/jamanetworkopen.2019.0766 (PMC6484656; doi:10.1001/jamanetworkopen.2019.0766)
Supplement: Supplement. — eTable 1. Self-Reported Risk Factors for Subsequent Administratively Recorded Suicide Attempts Involving History of Self-Injurious Thoughts and Behaviors Among Regular Army Soldiers Who Reported Lifetime Suicide Ideation in the STARRS Consolidated All Army Survey (n = 3,649) eTable 2. Self-Reported Risk Factors of Subsequent Administratively Recorded Suicide Attempts Involving Severity of Self-Injurious Thoughts and Behaviors Among Regular Army Soldiers Who Reported Lifetime Suicide Ideation in the STARRS Consolidated All Army Survey (n = 3,649) eTable 3. Self-Reported Risk Factors of Subsequent Administratively Recorded Suicide Attempts Involving History of Mental Disorders Among Regular Army Soldiers Who Reported Lifetime Suicide Ideation in the STARRS Consolidated All Army Survey (n = 3,649) eTable 4. Self-Reported Risk Factors of Subsequent Administratively Recorded Suicide Attempts Involving Socio-Demographics and Army Career Characteristics Among Regular Army Soldiers Who Reported Lifetime Suicide Ideation in the STARRS Consolidated All Army Survey (n = 3,649) eTable 5. Hyperparameter Settings for Super Learner Ensemble [file jamanetwopen-2-e190766-s001.pdf]

Supplementary Online Content

Zuromski KL, Bernecker SL, Gutierrez PM, et al. Assessment of a risk index for suicide attempts among US Army soldiers with suicide ideation: analysis of data from the Army Study to Assess Risk and Resilience in Servicemembers (Army STARRS). *JAMA Netw Open*. 2019;2(3):e190766. doi:10.1001/jamanetworkopen.2019.0766

eTable 1. Self-Reported Risk Factors for Subsequent Administratively Recorded Suicide Attempts Involving History of Self-Injurious Thoughts and Behaviors Among Regular Army Soldiers Who Reported Lifetime Suicide Ideation in the STARRS Consolidated All Army Survey (n = 3,649)
eTable 2. Self-Reported Risk Factors of Subsequent Administratively Recorded Suicide Attempts Involving Severity of Self-Injurious Thoughts and Behaviors Among Regular Army Soldiers Who Reported Lifetime Suicide Ideation in the STARRS Consolidated All Army Survey (n = 3,649)
eTable 3. Self-Reported Risk Factors of Subsequent Administratively Recorded Suicide Attempts Involving History of Mental Disorders Among Regular Army Soldiers Who Reported Lifetime Suicide Ideation in the STARRS Consolidated All Army Survey (n = 3,649)
eTable 4. Self-Reported Risk Factors of Subsequent Administratively Recorded Suicide Attempts Involving Socio-Demographics and Army Career Characteristics Among Regular Army Soldiers Who Reported Lifetime Suicide Ideation in the STARRS Consolidated All Army Survey (n = 3,649)
eTable 5. Hyperparameter Settings for Super Learner Ensemble

This supplementary material has been provided by the authors to give readers additional information about their work.

© 2019 Zuromski KL et al. *JAMA Network Open.*

eTable 1. Self-reported risk factors for subsequent administratively recorded suicide attempts involving history of self-injurious thoughts and behaviors among Regular Army soldiers who reported lifetime suicide ideation in the STARRS Consolidated All Army Survey (n = 3,649)[a]

	Distribution		Univariate 1[b]		Univariate 2		Univariate 3		Multivariate 1[c]		Multivariate 2[d]	
	%	(SE)	OR	(95% CI)	OR	(95% CI)	OR	(95% CI)	OR	(95% CI)	OR	(95% CI)
Ideation onset: ages 15-17 (vs. ≤14)	17.0	(1.6)	1.2	(0.4-3.6)					1.3	(0.4-4.3)	1.2	(0.4-3.7)
Ideation onset: ages 18+ (vs. ≤14)	43.8	(3.4)	1.0	(0.3-3.1)					0.7	(0.2-2.7)	0.7	(0.2-2.7)
F_2			0.1						0.4		0.2	
Years since onset of ideation (values=1-11+)	8.4	(0.2)	0.9	(0.8-1.1)					0.9	(0.8-1.1)	0.9	(0.8-1.1)
Active ideation (vs. passive)	77.1	(2.3)	2.1	(0.5-9.1)					0.9	(0.2-4.8)	1.5	(0.3-7.5)
Lifetime plan	39.4	(2.9)	3.4*	(1.5-7.9)					2.0	(0.8-4.9)		
Ideation recency: 30-day	11.8	(2.1)	8.5*	(3.3-22.0)					6.6*	(2.3-19.2)	8.5*	(3.3-22.0)
Lifetime attempt	19.7	(2.5)	4.0*	(1.7-9.3)	0.8	(0.1-6.8)						
Number of attempts (values=0-2+, mean)	0.3	(0.0)	2.6*	(1.5-4.4)	3.0	(0.8-11.5)	2.3	(0.8-6.7)	1.7	(0.9-3.2)		
Count of attempts: Exactly 1 (vs. 0)	12.7	(2.2)	2.3	(0.8-6.7)								
Count of attempts: 2+ (vs. 0)	7.0	(1.4)	6.8*	(2.1-22.3)								
F_2			5.0*									
Count of attempts: 2+ (vs. 0 or 1)	7.0	(1.4)					1.3	(0.2-11.7)				

*Significant at the .05 level, two-sided MI-adjusted test

[a]Results reflect weighted and multiply imputed (MI) data.

[b]The first univariate model included 2 predictors for ideation age-of-onset, 1 for years since ideation age-of-onset, and 1 for active (vs. passive) ideation. Each univariate OR presented thereafter controlled for these 4 predictors .

[c]Multivariate model 1 carried forward the 4 controls and significant predictors from the univariate analysis.

[d]Multivariate model 2 dropped the non-significant predictors from multivariate model 1.

© 2019 Zuromski KL et al. *JAMA Network Open.*

eTable 2. Self-reported risk factors of subsequent administratively recorded suicide attempts involving severity of self-injurious thoughts and behaviors among Regular Army soldiers who reported lifetime suicide ideation in the STARRS Consolidated All Army Survey (n = 3,649)[a]											
	**Distribution**			**Univariate**			**Multivariate 1[b]**			**Multivariate 2[c]**	
	**%**	**(SE)**		**OR**	**(95% CI)**		**OR**	**(95% CI)**		**OR**	**(95% CI)**
Severity of suicidal ideation during worst week											
Days per week (vs. 1)											
2	13.8	(1.7)		1.3	(0.2-8.9)						
3	14.7	(1.7)		2.2	(0.2-15.1)						
4	9.9	(1.6)		1.5	(0.2-10.2)						
5	7.9	(0.9)		1.5	(0.2-10.2)						
6	5.5	(0.9)		0.4	(0.0-4.8)						
7	23.3	(2.6)		4.1	(1.0-17.9)						
F_6				1.5							
7 (vs. 6 or less)	23.3	(2.6)		3.0*	(1.2-7.7)		2.1	(0.7-6.1)			
Time per day (vs. just a few moments)											
Less than 1 hour	26.6	(2.2)		1.7	(0.3-9.3)						
1 to 4 hours	28.9	(1.9)		1.8	(0.3-10.7)						
5 to 8 hours	11.0	(1.7)		2.8	(0.6-13.1)						
9+ hours	11.2	(2.0)		6.4*	(1.5-27.3)						
F_6				2.2							
9+ hours (vs. ≤8 hours)	11.2	(2.0)		3.6*	(1.4-9.1)		2.2	(0.8-6.5)			
Worst week severity (vs. neither)											
7 days per week OR 9+ hours per day	17.7	(1.7)		2.0	(0.7-6.1)					1.7	(0.5-5.4)
7 days per week AND 9+ hours per day	8.4	(1.9)		4.8*	(1.7-13.7)					3.8*	(1.1-12.5)
F_2				4.0*						2.3	
7 days per week AND/OR 9+ hours per day (vs. neither)	26.1	(1.8)		3.0*	(1.2-7.7)						
Controllability of thoughts (vs. easy)											
A little difficult	32.1	(2.2)		1.6	(0.3-8.3)						
Somewhat difficult	19.1	(2.5)		2.6	(0.6-11.7)						
Very difficult	16.8	(1.9)		3.9	(0.8-19.9)						
Impossible	5.2	(1.1)		5.6	(1.0-31.4)						
F_4				1.1							

© 2019 Zuromski KL et al. *JAMA Network Open.*

Very difficult to impossible (vs. somewhat difficult or less)	22.0	(1.3)		1.5	(0.5-4.5)						
Any difficulty (vs. easy/no difficulty)	73.2	(1.1)		1.8	(0.4-7.6)						
Frequency of tempting fate (reference: never)											
Rarely	28.4	(3.0)		2.2	(0.6-7.8)						
Sometimes	14.2	(1.5)		2.5	(0.7-9.3)						
Often	10.0	(1.3)		2.9	(0.9-9.6)						
F_3				1.3							
Sometimes/often (vs. rarely/never)	24.2	(1.4)		1.8	(0.6-5.4)						
Ever (vs. never)	52.6	(1.6)		2.5	(0.9-6.6)						
Lifetime presence of non-suicidal self-injury	25.9	(2.7)		1.6	(0.7-3.8)						

*Significant at the .05 level, two-sided MI-adjusted test

[a]Results reflect weighted and multiply imputed (MI) data. All models controlled for ideation age-of-onset, years since ideation age-of-onset, active (vs. passive) ideation, and 30-day ideation recency (as defined in eTable 1) and time-varying rank (as defined in eTable 4).

[b]Multivariate model 1 included significant predictors from univariate models.

[c]Multivariate model 2 dropped the non-significant predictors from multivariate model 1.

© 2019 Zuromski KL et al. *JAMA Network Open.*

eTable 3. Self-reported risk factors of subsequent administratively recorded suicide attempts involving history of mental disorders among Regular Army soldiers who reported lifetime suicide ideation in the STARRS Consolidated All Army Survey (n = 3,649)[a]

	Distribution			Univariate 1			Multivariate 1[b]	
	%	(SE)		OR	(95% CI)		OR	(95% CI)
**Mental disorders**								
Internalizing								
Major depressive episode	72.2	(2.0)		4.5	(0.3-39.8)		2.3	(0.2-24.6)
Generalized anxiety disorder	59.7	(2.5)		2.2	(0.8-5.9)		0.9	(0.3-2.7)
Post-traumatic stress disorder	65.5	(2.3)		6.8*	(1.1-40.1)		4.6	(0.6-32.6)
Bipolar disorder	8.7	(1.2)		2.4	(0.8-7.2)		1.9	(0.6-6.2)
Panic disorder	11.1	(1.6)		0.8	(0.3-1.8)		0.5	(0.2-1.2)
Any	88.8	(1.6)		34.1*	(8.5-137.3)			
$F_{5/4}$							1.6	
Externalizing								
Intermittent explosive disorder	42.7	(2.0)		1.1	(0.5-2.8)		0.8	(0.3-1.9)
Attention-deficit/hyperactivity disorder	15.6	(2.4)		2.9*	(1.2-6.9)		2.5	(1.0-6.6)
Substance use disorder	27.9	(2.0)		2.0	(0.7-5.4)		1.5	(0.5-4.5)
Any	53.3	(1.9)		5.3*	(1.3-21.4)			
F_3							1.4	
F_8							1.4	
Total								
Any mental disorder	92.3	(1.1)		51.6*	(6.2-427.1)			
Summary measure 1								
Count of mental disorders: Exactly 1 or 2 (vs. 0)	29.5	(1.4)		24.0*	(1.6-351.1)			
Count of mental disorders: 3+ (vs. 0)	62.8	(1.2)		62.9*	(7.8-506.5)			
F_2				8.1*				
Summary measure 2								
Count of mental disorders: 2+ (vs. 0 or 1)	79.6	(1.5)		32.5*	(7.9-134.3)			
Summary measure 3								
Count of mental disorders: Exactly 1 (vs. 0)	12.8	(1.5)		2.4	(0.2-33.0)			
Count of mental disorders: Exactly 2 (vs. 0)	16.8	(1.6)		39.9*	(2.6-621.9)			

© 2019 Zuromski KL et al. *JAMA Network Open.*

Count of mental disorders: Exactly 3 (vs. 0)	24.7	(2.5)		33.7*	(3.5-328.1)			
Count of mental disorders: Exactly 4 (vs. 0)	19.2	(2.1)		61.8*	(6.9-556.0)			
Count of mental disorders: 5+ (vs. 0)	18.9	(1.8)		99.2*	(11.7-843.3)			
$F_{(3,172)}$				6.0*				

*Significant at the .05 level, two-sided MI-adjusted test

[a]Results reflect weighted and multiply imputed (MI) data. All models controlled for ideation age-of-onset, years since ideation age-of-onset, active (vs. passive) ideation, and 30-day ideation recency (as defined in eTable 1) and time-varying rank (as defined in eTable 2).

[b]Multivariate model 1 entered all 8 mental disorders as predictors in the model.

© 2019 Zuromski KL et al. *JAMA Network Open.*

eTable 4. Self-reported risk factors of subsequent administratively recorded suicide attempts involving socio-demographics and Army career characteristics among Regular Army soldiers who reported lifetime suicide ideation in the STARRS Consolidated All Army Survey (n = 3,649)[a]

	Distribution			Univariate			Multivariate 1	
	%	(SE)		OR	(95% CI)		OR	(95% CI)
**Socio-demographics**								
Age (mean, in decades)	3.1	(0.0)		0.5*	(0.3-1.0)		1.3	(0.4-4.5)
Sex: female (vs. male)	19.5	(1.7)		0.9	(0.3-2.6)			
Race (vs. Non-Hispanic white)								
Non-Hispanic black	14.6	(1.7)		2.5	(0.8-7.8)			
Hispanic	9.0	(1.1)		0.3	(0.1-1.5)			
Other	7.0	(1.2)		0.4	(0.0-3.9)			
F_3				2.3				
Marital history (vs. currently)								
Previously	9.2	(1.6)		0.7	(0.2-2.7)			
Never	25.1	(2.6)		0.9	(0.3-2.6)			
F_2				0.2				
**Army career characteristics**								
Current years of service (means, in decades)	0.9	(0.0)		0.4*	(0.2-0.9)		1.0	(0.2-4.0)
MOS (vs. combat service support)								
Combat arms	34.5	(2.2)		0.7	(0.3-1.5)			
Combat support	22.5	(2.1)		0.5	(0.1-2.1)			
F_2				0.7				
Rank (vs. officer)								
Junior	32.9	(2.8)		33.7*	(3.4-285.6)		42.6*	(2.8-649.7)
Senior	45.4	(2.9)		8.3*	(1.0-68.5)		9.4	(0.9-94.4)
F_2				7.6*			4.3*	
Deployment (vs. never)								
Currently	9.6	(1.6)		0.5	(0.1-3.1)			
Previously	69.8	(2.8)		1.0	(0.4-2.7)			
F_2				0.4				

*Significant at the .05 level, two-sided MI-adjusted test

[a]Results reflect weighted and multiply imputed (MI) data. All models controlled for ideation age-of-onset, years since ideation age-of-onset, active (vs. passive) ideation, and 30-day ideation recency (as defined in eTable 1) and time-varying rank (as defined in eTable 2)

eTable 5. Hyperparameter settings for Super Learner ensemble[a]					
	External fold weights				
	1	2	3	4	5
Generalized linear models[b]					
Screener = p<.1	0.03056	0.02668	0.02473	0.02272	0.01664
Screener = LASSO, Minimum 5	0.03056	0.02474	0.02473	0.02389	0.01664
Screener = All	0.02993	0.02839	0.02552	0.02274	0.01664
Elastic net penalized regression[c]					
Alpha = 0	0.02993	0.02839	0.02549	0.02387	0.01731
Alpha = 0.1	0.03076	0.02839	0.02549	0.02271	0.01731
Alpha = 0.2	0.03090	0.02839	0.02549	0.02387	0.01731
Alpha = 0.3	0.03090	0.02839	0.02549	0.02269	0.01731
Alpha = 0.4	0.03076	0.02839	0.02579	0.02269	0.01731
Alpha = 0.5	0.03076	0.02839	0.02549	0.02271	0.01731
Alpha = 0.6	0.03076	0.02839	0.02549	0.02389	0.01731
Alpha = 0.7	0.03076	0.02839	0.02549	0.02271	0.01731
Alpha = 0.8	0.03076	0.02839	0.02549	0.02269	0.01731
Alpha = 0.9	0.03076	0.02839	0.02473	0.02269	0.01731
Generalized Additive Models[d]					
Target degrees of freedom = 3	0.02836	0.02839	0.02530	0.02710	0.01741
Target degrees of freedom = 4	0.02836	0.03010	0.02515	0.02822	0.01741
Target degrees of freedom = 5	0.02836	0.03010	0.02515	0.02813	0.01741
Target degrees of freedom = 6	0.02836	0.02795	0.02500	0.03001	0.01741
Linear multivariate adaptive regression splines[e]					
Screener p<.1	0.05596	0.09909	0.01135	0.07711	0.12819
Screener LASSO, minimum 5	0.00000	0.08240	0.06152	0.01091	0.01315
Random Forests[f]					
ntree = 8000	0.03883	0.05118	0.02608	0.11847	0.11051
ntree = 10000	0.04602	0.04216	0.04328	0.11542	0.11256
Support Vector Machines[g]					
Radial kernel, Cost = 1,000	0.01982	0.00000	0.04910	0.00017	0.00000
Radial kernel, Cost = 1,500	0.02735	0.00000	0.01004	0.00022	0.00814
Polynomial kernel, Cost = 1,000, Coef() = 2	0.07483	0.04146	0.05961	0.02964	0.00874
Polynomial kernel, Cost = 1,000, Coef() = 4	0.03521	0.05341	0.05045	0.08302	0.07118
Polynomial kernel, Cost = 10,000, Coef() = 2	0.06961	0.00024	0.04763	0.06303	0.03083
Polynomial kernel, Cost = 10,000, Coef() = 4	0.01430	0.06869	0.05434	0.05545	0.04805
Bayesian Additive Regression Trees[h]					
Number of trees = 25	0.02074	0.00000	0.01310	0.02291	0.00888
Number of trees = 50	0.02635	0.00000	0.00000	0.00088	0.01825
Number of trees = 75	0.00000	0.02709	0.05672	0.00044	0.08347
Regularized gradient boosting[i]					
Number of trees = 30,000	0.02978	0.02143	0.03800	0.00237	0.03268
Number of trees = 40,000	0.02986	0.03258	0.04850	0.00664	0.03268

[a]The Super Learner optimization method used 1 - cvAUC as the loss function to be optimized. This method returns low non-zero weights rather than zero weights for poor-performing classifiers. As a result, Super Learner usually performs better when poor-performing classifiers are not included in the library. Based on this fact, we excluded the following classifiers because of preliminary evidence of

© 2019 Zuromski KL et al. *JAMA Network Open.*

poor performance in a larger original library: polynomial multivariate adaptive regression splines, neural networks, and support vector machines with a linear kernel.

[b]McCullagh P, Nelder JA. *Generalized Linear Models*. 2nd ed. Champan & Hall/CRC Monographs on Statistics & Applied Probability Series, #37. Taylor & Francis; 1989.
[c]Zou H, Hastie T. Regularization and variable selection via the elastic net. *J R Stat Soc Series B Stat Methodol.* 2005;67(2):301-320. doi:10.1111/j.1467-9868.2005.00503.x
[d]Hastie TJ, Tibshirani RJ. *Generalized Additive Models*. 1st ed. Chapman & Hall/CRC Monographs on Statistics and Applied Probability, #43; 1990.
[e]Friedman JH. Multivariate adaptive regression splines. *The Annals of Statistics.* 1991;19(1):1-67. doi:10.1214/aos/1176347963
[f]Breiman L. Random forests. *Machine Learning.* 2001;45(1):5-32. doi:10.1023/a:1010933404324
[g]Steinwart I, Christmann A. *Support Vector Machines*. New York, NY: Springer-Verlag New York; 2008.
[h]Chipman H, George EI, McCulloch R. BART: Bayesian additive regression trees. *Ann Appl Stat.* 2010;4(1):266-298. doi:10.1214/09-aoas285

© 2019 Zuromski KL et al. *JAMA Network Open.*
